# The Role of Cardiac Troponin T Quantity and Function in Cardiac Development and Dilated Cardiomyopathy

**DOI:** 10.1371/journal.pone.0002642

**Published:** 2008-07-09

**Authors:** Ferhaan Ahmad, Sanjay K. Banerjee, Michele L. Lage, Xueyin N. Huang, Stephen H. Smith, Samir Saba, Jennifer Rager, David A. Conner, Andrzej M. Janczewski, Kimimasa Tobita, Joseph P. Tinney, Ivan P. Moskowitz, Antonio R. Perez-Atayde, Bradley B. Keller, Michael A. Mathier, Sanjeev G. Shroff, Christine E. Seidman, J. G. Seidman

**Affiliations:** 1 Cardiovascular Institute, Department of Medicine, University of Pittsburgh, Pittsburgh, Pennsylvania, United States of America; 2 Department of Human Genetics, University of Pittsburgh, Pittsburgh, Pennsylvania, United States of America; 3 Department of Genetics, Howard Hughes Medical Institute and Harvard Medical School, Boston, Massachusetts, United States of America; 4 Department of Bioengineering, University of Pittsburgh, Pittsburgh, Pennsylvania, United States of America; 5 Department of Pediatrics, Children's Hospital of Pittsburgh, University of Pittsburgh, Pittsburgh, Pennsylvania, United States of America; 6 Department of Pathology, Children's Hospital, Boston, Massachusetts, United States of America; University of Cincinnati, United States of America

## Abstract

**Background:**

Hypertrophic (HCM) and dilated (DCM) cardiomyopathies result from sarcomeric protein mutations, including cardiac troponin T (cTnT, *TNNT2*). We determined whether *TNNT2* mutations cause cardiomyopathies by altering cTnT function or quantity; whether the severity of DCM is related to the ratio of mutant to wildtype cTnT; whether Ca^2+^ desensitization occurs in DCM; and whether absence of cTnT impairs early embryonic cardiogenesis.

**Methods and Findings:**

We ablated *Tnnt2* to produce heterozygous *Tnnt2* ^+/−^ mice, and crossbreeding produced homozygous null *Tnnt2* ^−/−^ embryos. We also generated transgenic mice overexpressing wildtype (TG^WT^) or DCM mutant (TG^K210Δ^) *Tnnt2*. Crossbreeding produced mice lacking one allele of *Tnnt2*, but carrying wildtype (*Tnnt2* ^+/−^/TG^WT^) or mutant (*Tnnt2* ^+/−^/TG^K210Δ^) transgenes. *Tnnt2* ^+/−^ mice relative to wildtype had significantly reduced transcript (0.82±0.06[SD] vs. 1.00±0.12 arbitrary units; *p* = 0.025), but not protein (1.01±0.20 vs. 1.00±0.13 arbitrary units; *p* = 0.44). *Tnnt2* ^+/−^ mice had normal hearts (histology, mass, left ventricular end diastolic diameter [LVEDD], fractional shortening [FS]). Moreover, whereas *Tnnt2* ^+/−^/TG^K210Δ^ mice had severe DCM, TG^K210Δ^ mice had only mild DCM (FS 18±4 vs. 29±7%; *p*<0.01). The difference in severity of DCM may be attributable to a greater ratio of mutant to wildtype *Tnnt2* transcript in *Tnnt2* ^+/−^/TG^K210Δ^ relative to TG^K210Δ^ mice (2.42±0.08, *p* = 0.03). *Tnnt2* ^+/−^/TG^K210Δ^ muscle showed Ca^2+^ desensitization (pCa_50_ = 5.34±0.08 vs. 5.58±0.03 at sarcomere length 1.9 µm, *p*<0.01), but no difference in maximum force generation. Day 9.5 *Tnnt2* ^−/−^ embryos had normally looped hearts, but thin ventricular walls, large pericardial effusions, noncontractile hearts, and severely disorganized sarcomeres.

**Conclusions:**

Absence of one *Tnnt2* allele leads to a mild deficit in transcript but not protein, leading to a normal cardiac phenotype. DCM results from abnormal function of a mutant protein, which is associated with myocyte Ca^2+^ desensitization. The severity of DCM depends on the ratio of mutant to wildtype *Tnnt2* transcript. cTnT is essential for sarcomere formation, but normal embryonic heart looping occurs without contractile activity.

## Introduction

The long-term response of the heart to pathological stimuli is composed of maladaptive remodeling characterized by hypertrophy and/or dilation, leading to heart failure. Individuals in the United States are at a 20% lifetime risk of heart failure, which is the most common cause of death. Cardiomyopathies are primary disorders of the myocardium resulting from heritable mutations in single genes. Familial hypertrophic (HCM) and dilated cardiomyopathy (DCM) are among the most common inherited cardiovascular disorders, with prevalences of 200 and 36.5/100,000, respectively [1]. At least 70% of HCM is caused by mutations in sarcomeric protein genes. The cardiac troponin T protein (cTnT), encoded by the gene *TNNT2*, is a component of the troponin complex which allows actomyosin interaction and contraction to occur in response to Ca^2+^. Although *TNNT2* is commonly mutated in HCM, surprisingly, it has been found that distinct *TNNT2* mutations also lead to DCM [2].

Cardiomyopathies secondary to *TNNT2* mutations are inherited as autosomal dominant traits. In autosomal dominant diseases, only one of two alleles of the responsible gene is mutant. In some instances, mutations produce disease by inactivating an allele and reducing the quantity of functional protein (haploinsufficiency). However, in other instances, mutations create a mutant protein (“poison peptide”) which interferes with normal function or assumes a new function. A few mouse models have been reported with ablations of other genes encoding sarcomeric proteins mutated in HCM. In the heterozygous state, in which one allele remains intact, no phenotype abnormalities have been noted for α tropomyosin [3], [4], cardiac myosin binding protein C [5], and cardiac troponin I [6]. These observations suggest that haploinsufficiency of these genes does not lead to HCM. However, no ablations have been studied of a gene mutated with a significant frequency in both HCM and DCM. The differing phenotypes of HCM and DCM resulting from *TNNT2* mutations suggests that divergent mechanisms lead from different mutations to either phenotype. To address whether haploinsufficiency of *TNNT2* is partially or completely responsible for either HCM or DCM, we ablated *Tnnt2* in a mouse model by gene targeting.

Since haploinsufficiency is unlikely to lead to both HCM and DCM, it is probable that abnormal function of a mutated cTnT contributes to at least one phenotype. To assess possible “poison peptide” *in vivo* effects of the human Lys210 deletion (K210Δ) DCM mutation in *TNNT2*, we generated transgenic mice with cardiac overexpression of mutant or wildtype cTnT. Moreover, we determined the effect of the relative abundance of mutant cTnT on the severity of DCM. Although previous investigations have been inconsistent, *in vitro* studies have suggested that HCM mutations in several genes lead to an increase in Ca^2+^ sensitivity, increases in tension generation, and/or increases in ATPase activity [7]–[12], whereas DCM mutations are associated with Ca^2+^ desensitization and/or decreased ATPase activity *in vitro* or in permeabilized rabbit cardiac muscle fibers and isolated myocytes [13]–[18].

In this study, heterozygous mice lacking one allele of *Tnnt2* (*Tnnt2* ^+/−^) had a mild deficit in transcript, no detectable deficit in protein, and no detectable phenotype abnormalities. In contrast, homozygous null embryos (*Tnnt2* ^−/−^) had disorganized sarcomeres and noncontractile hearts leading to death by embryonic day 10.5. Despite the absence of contractile activity, normal cardiac looping occurred in these embryos. Moreover, when a transgene with the human DCM *TNNT2* K210Δ mutation was introduced into *Tnnt2* ^+/−^ mice (*Tnnt2* ^+/−^/TG^K210Δ^), they developed DCM and their papillary muscle fibers showed Ca^2+^ desensitization. TG^K210Δ^ mice, with two endogenous alleles of *Tnnt2* intact, had a lower ratio of mutant to wildtype transcript, and a milder phenotype. These results suggest that *TNNT2* mutations lead to cardiomyopathies because of abnormally functioning mutant cTnT rather than haploinsufficiency of the protein. Moreover, the severity of DCM is correlated with the ratio of mutant to wildtype *Tnnt2* transcript. Although cTnT is essential for sarcomere formation and contraction, normal looping of the embryonic heart occurs in the absence of contractile activity.

## Materials and Methods

All studies were approved by the University of Pittsburgh Institutional Animal Care and Use Committee (IACUC). All mice were studied at 8–10 weeks of age unless otherwise indicated.

### Generation of Tnnt2 ablated mice

**Figure 1:**
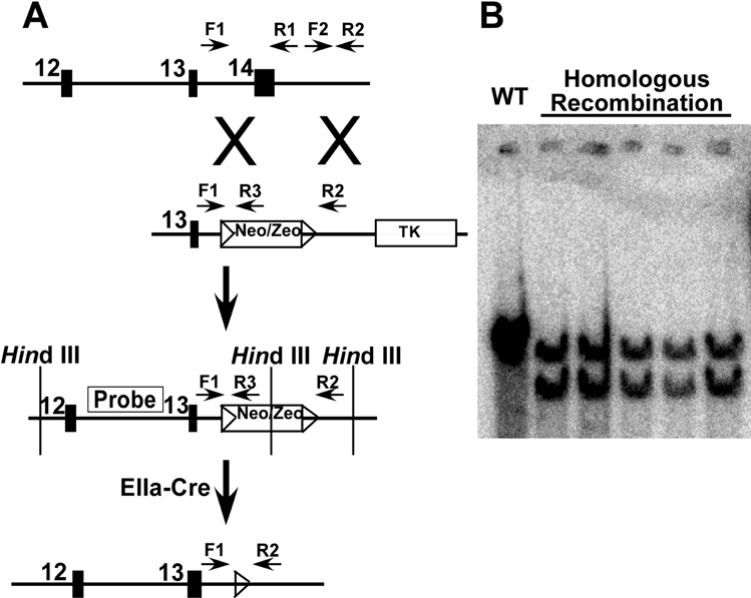
Generation of *Tnnt2* ^+/^^−^ mice. A. A targeting construct containing a neomycin resistance gene (*neo/zeo*) between *lox*P sites (triangles) was introduced into the murine *Tnnt2* locus by homologous recombination in murine ES cells, ablating the 3′ segment of the gene, including exon 14. ES cells were microinjected into mouse blastocysts to generate chimeras, which were bred with wildtype mice for germline transmission of the *Tnnt2* ablation. The neomycin resistance gene was excised using Cre-mediated excision by mating with EIIa-Cre recombinase mice. Horizontal arrows indicate PCR primers (F1, F2, R1, R2, R3) used for genotyping as described in “Materials and Methods.” B. Genomic DNA from mice prior to Cre-mediated excision of the neomycin resistance gene was digested with *Hin*d III and Southern blotted with the probe indicated in panel A to demonstrate homologous recombination. A *Hin*d III restriction site in the neomycin resistance gene produced a smaller restriction product in the presence of homologous recombination. WT, wildtype; TK, thymidine kinase gene.

A murine genomic segment containing the *Tnnt2* gene was subcloned from the CitbCJ7 BAC library, clone 353I2 (Invitrogen). A *lox*P-flanked (floxed) neomycin resistance gene driven by the *PGK* promoter was inserted in place of the 3′ segment of *Tnnt2*, including exon 14, with a thymidine kinase gene downstream of the genomic segment (Figure 1) [19]. The construct was electroporated into 129/SvEv strain TC1 ES cells (a kind gift from Philip Leder, M.D., Harvard Medical School). Targeted ES cells were selected with Genetecin/G418 (Invitrogen) and FIAU (Moravek), and homologous recombination confirmed by Southern blotting. ES cells were microinjected into mouse blastocysts to generate chimeras, which were then bred to produce heterozygotes with *Tnnt2* ablation (*Tnnt2* ^+/−^). Genotypes were confirmed by Southern blotting and multiplex PCR using three primers to amplify the wildtype and mutant alleles (primers F1, 5′-ATGACAACCAGAAAGTGTGAGTGT-3′; R1, 5′-GAGTTGGACAGATACAAGGGTCTT-3′; R3, 5′-CTGGACGTAAACTCCTCTTCAGAC-3′). With *Tnnt2* ablation, primers F1 and R3 gave a product size of 260 bp; in the presence of wildtype genomic sequence, F1 and R1 gave a product size of 617 bp. The floxed neomycin cassette was excised by mating with transgenic EIIa-Cre recombinase mice (129/SvEv background). Genotyping was performed by multiplex PCR using three primers designed to amplify the wildtype and mutant alleles (primers F1; F2, 5′-GCTGTATTTCACATCCAAACCATA-3′; R2, 5′-TCCTGGTGACTGATGATAATAACG-3′). With the neomycin cassette excised, primers F1 and R2 gave a product size of 324 bp; in the presence of wildtype genomic sequence, F2 and R2 gave a product size of 506 bp.

### Generation of Tnnt2 transgenic mice

The *Tnnt2* cDNA was amplified from murine cardiac RNA by reverse transcription-polymerase chain reaction (RT-PCR) (Qiagen) with primers F3, 5′- AGACCTGTGTCGACTCCCTGTTCAGAGGGAGAGCCGAGAG-3′, and R4, 5′- AAACAGGAGTAAGCTTTGGGTGCCAAGGAGGACCCAGAGC-3′, and then subjected to PCR mutagenesis to delete nucleotides AAG at positions 628–630, encoding the lysine deletion at codon 210 (K210Δ). The wildtype and K210Δ *Tnnt2* cDNA were inserted into a pC126 expression vector, containing a highly active cardiac myocyte specific αMHC promoter, a human growth hormone 3′ untranslated region (UTR), and a polyadenylation terminator (a kind gift from Jeffrey Robbins, Ph.D., Cincinnati Children's Hospital) [20]. The plasmids were linearized, size fractionated, purified (QIAquick Gel Extraction Kit, Qiagen), and microinjected into fertilized FVB mouse oocytes. Presence of the wildtype (TG^WT^) and K210Δ *Tnnt2* (TG^K210Δ^) transgenes was confirmed by Southern blotting and PCR with primers F4, 5′-CTGAGACAGAGGAGGCCAAC-3′, and R5, 5′-CAGCCTCCAGGTTGTGAATA-3′. Five TG^K210Δ^ and four TG^WT^ founder lines were generated, and lines of each genotype had similar phenotypes. One representative mutant and wildtype transgenic line was backcrossed for at least ten generations into a 129/SvEv background to generate TG^K210Δ^, TG^WT^, *Tnnt2* ^+/−^/TG^K210Δ^ and *Tnnt2* ^+/−^/TG^WT^ mice in a uniform genetic background.

### RNA analyses

Hearts were harvested immediately after sacrifice of the mouse, washed in PBS, and flash frozen in liquid nitrogen. RNA was isolated from cardiac tissue by lysis in ice cold Trizol (Invitrogen) and chloroform extraction and treated with 10 U DNase I (Roche) / 10 µg at 37°C for 10 min. RNA quantity was determined by OD measurement at 260 nm. Northern blots were performed with 2 µg RNA per gel lane using the following antisense biotinylated riboprobes (Strip-EZ RNA Kit, Ambion): the *Tnnt2* coding sequence, corresponding to nucleotides 169–630 of the published sequence (NM_011619); the *Tnnt2* 3′ UTR (present only in endogenous gene transcript), corresponding to nucleotides 909–1111 of the published sequence (NM_011619); the human growth hormone 3′ UTR (present only in transgene transcript); or the GAPDH coding sequence. Reverse transcription-PCR was performed and the product sequenced using the BigDye Terminator v3.1 Cycle Sequencing Kit (ABI).

Realtime quantitative reverse transcription-PCR (QPCR) was performed as described [21]. Reverse transcription was carried out with the SuperScript First-Strand Synthesis System as recommended by the manufacturer (Invitrogen). The cDNA was then used as template for QPCR with specific primers on an ABI Prism 7000 Sequence Detection System (Applied Biosystems). Primers amplifying the housekeeping gene cyclophilin were used as a control. Hot-start PCR was performed with the SYBR Green PCR Master Mix (Applied Biosystems). The PCR mixtures were pre-heated at 50°C for 2 min and then at 95°C for 10 min to activate the AmpliTaq Gold DNA polymerase, followed by 40 cycles of amplification (95°C for 15 s; 60°C for 1 min). A final extension step was performed at 60°C for 10 min. Primers were tested on cDNA, reverse transcriptase-negative samples, and 0.1% diethyl pyrocarbonate-treated water to exclude amplification of genomic DNA and primer-primer interactions. Equivalence and efficiency were tested by amplifications on serial dilutions of RNA. Quantification was performed using the comparative Ct method (2^−ΔΔCt^).

### Protein analyses

Cardiac tissue was homogenized in 20 volumes of protein extraction buffer (in mM, 50 Tris at pH 8.0, 200 NaCl, 20 NaF, 20 β-glycerolphosphate, 1 DTT, with 0.5% NP40, 1 protease inhibitor tablet / 7 ml buffer, and 1 phosphatase inhibitor tablet / 10 ml buffer [Roche]). The homogenate was allowed to settle on ice for 10 min, and then centrifuged at 10,000 × g for 10 min. The supernatant was stored at -80°C for protein studies. All protein quantities in the proposed studies were measured by the Bradford method (Bio-Rad). Immunoblots were performed a using 10–20 µg of protein per lane loaded onto an appropriate concentration protein gel (Pierce) and subjected to electrophoresis at 120 V for 45 min. Samples were transferred from the gel to a PVDF membrane. The membrane was blocked and incubated at 4°C overnight with primary antibody recognizing cTnT, troponin C (TnC), troponin I (cTnI), actin, tropomyosin, β-myosin heavy chain (MHC), or GAPDH (Santa Cruz Biotechnology, # sc-8121, #sc-20642, #sc-15368, #sc-1615, #sc-18174, #sc-20641, and RDI Research Diagnostics # RDI-TRK5G4-6C5, respectively) at 1∶200 dilution, followed by the appropriate horseradish peroxidase-conjugated secondary antibody (Santa Cruz or Amersham) at 1∶5000 dilution at room temperature for 45 min. Proteins were visualized and quantified by enhanced chemiluminescence (Pierce). Signal intensity was normalized to protein loading using GAPDH immunoblots or Coomassie blue gel staining.

### Echocardiography

Transthoracic echocardiography was performed on a VisualSonics Vevo 770 machine [22]. Mice were sedated with tribromoethanol (125 mg/kg IP). 2D short-axis images of the left ventricle were obtained. Left ventricular end diastolic (LVEDD) and end systolic (LVESD) chamber dimensions and wall thickness (LVWT) were obtained from M-mode tracings based on measurements averaged from three separate cardiac cycles. Left ventricular fractional shortening (FS) was calculated as (LVEDD-LVESD)/ LVEDD × 100%.

### Electrocardiography (ECG) and electrophysiological studies

Continuous telemetry electrocardiograms (ECGs) were recorded from conscious mice and analyzed as described [23]. Mice were anesthetized with tribromoethanol, wireless transmitters (Data Sciences International) implanted subcutaneously, and leads tunneled subcutaneously to the right shoulder and the left subcostal areas to simulate a lead II surface ECG. For monitoring, mice were housed in cages over receiver plates connected to a computer where digitized signals were stored. Monitoring was performed for ≥24 hours. Data were analyzed for heart rate, cardiac cycle intervals, and spontaneous atrial or ventricular arrhythmia occurrence.

*In vivo* electrophysiological studies were performed as described [24]. An ECG was obtained with subcutaneous limb needles. An octapolar catheter (NuMed) was advanced through the right external jugular vein to the right ventricular apex. Baseline cardiac cycle intervals were measured including RR, PR, QRS, QT, AH, and HV intervals. Sinus node function was evaluated by measuring sinus node recovery time (SNRT) after pacing the right atrium at a cycle length of 100 ms for 60 s, and correcting for baseline heart rate (SNRTc = SNRT−RR) (Bloom stimulator, Fisher). Atrioventricular (AV) and VA Wenckebach and 2∶1 cycle lengths were determined. Programmed atrial and ventricular stimulation was performed by delivering a premature stimulus after the eighth stimulus in a drive train. Effective (ERP) and functional refractory periods (FRP) were determined at a cycle length of 100 ms for both AV and VA conduction. Ventricular ERP was determined at a drive cycle length of 100 ms. Programmed ventricular stimulation, consisting of burst pacing at cycle lengths of 100 ms to 50 ms in decrements of 10 ms, and of programmed stimulation with double and triple extrastimuli at a drive cycle length of 100 ms with a coupling interval ≥30 ms, were performed. Inducibility was defined as 10 beats of ventricular tachycardia (VT).

### Histopathology

For light microscopy, cardiac tissue was fixed in 4% formaldehyde (Polysciences), mounted in paraffin blocks, and sections stained with hematoxylin and eosin (H&E) or Masson trichrome. For electron microscopy, tissue was fixed with 2.5% gluteraldehyde and 2% paraformaldehyde in 0.11 M cacodylate buffer, postfixed in 1% osmium tetroxide (OsO_4_), and then 1% uranyl acetate. The fixed tissue was dehydrated in alcohol, rinsed in propylene oxide, and embedded with 1∶1 propylene and Spurr's resin solution. The solution was replaced with 100% Spurr's solution and polymerized at 70°C for 48 hours.

### Biomechanical studies

Hearts were excised, placed in ice-cold PBS, and then transferred to relaxing solution (in mM, 40 BES [pH 7.0], 20 KCl, 1 free Mg^2+^, 5 MgATP, 10 creatine phosphate, 20 EGTA, 1 DTT, 0.01 leupeptin, 0.1 PMSF, 180 ionic strength). Anterior left ventricular papillary muscles were dissected, cut into strips, and skinned overnight in relaxing solution with 1% Triton X-100 at 4°C. Skinned muscle bundles were mounted in a 750 µl bath and were attached to a length controller (model 322B, Aurora Scientific, Toronto, Ontario, Canada) at one end and a force transducer (model 403A, Aurora Scientific, Toronto, Ontario, Canada) at the other end using aluminum T-clips (KEM-MIL-CO, Sunnyvale, CA). Average bundle dimensions were approximately 1 mm long by 180 µm wide. Sarcomere length (SL) was determined using laser diffraction (Spectra-Physics, 10mW HeNe laser, Mountain View, CA) and set at either 1.9 or 2.3 µm. The muscle bundle was then fully activated twice prior to generation of force-pCa curves. Activating solution (pCa 4.33) contained all the ingredients of relaxing solution (pCa 10). Force-pCa data were collected by exposing the skinned fiber to various concentrations of free calcium (pCa range 7.00–4.33) that were generated by mixing relaxing and activating solutions in appropriate proportions.

Normalized force was calculated as the ratio of the measured force at a given pCa and the maximally activated force (i.e., force at pCa = 4.33). Normalized force-pCa data were fitted to a modified Hill equation [25] using a nonlinear regression algorithm (Prism, GraphPad Software, San Diego, CA). Two parameters were estimated from normalized force-pCa data for each fiber: pCa_50_ (pCa required to produce normalized force of 50%) and Hill coefficient (a measure of the steepness of the normalized force-pCa curve, which characterizes the cooperative phenomena in muscle force generation). All quantitative data are presented in tables as mean ± standard deviation (SD) or mean ± standard error (SE), as indicated. Most of the analysis consisted of comparing two groups. This was accomplished using either Student's *t* test or chi-squared test. The analysis of force-pCa data consisted of more than two groups: skinned muscle fibers from two types of mice, with measurements made at two sarcomere lengths in each muscle fiber. These data were analyzed using two-way (mouse type and sarcomere length) ANOVA with one repeated measure (sarcomere length). *Post hoc* pairwise comparisons were made using the Tukey's test.

### Embryonic studies

Three to four week old *Tnnt2* ^+/−^ females were superovulated with pregnant mare serum (5 IU IP), followed two days later by human chorionic gonadotropin (5 IU IP), and then mated with adult *Tnnt2* ^+/−^ males. Pregnant females were sacrificed and embryos were harvested at 8.5–12.5 days postcoitum (pc). For qualitative studies of contractility, day 9.5 embryos were attached to the experimental chamber on the stage of an Olympus IX71 inverted microscope. The chamber was perfused with 3 ml/min Tyrode solution at 37°C. Cardiac contractile function was indexed by spontaneous or electrically stimulated heartbeats measured with a video edge detector and specialized data acquisition software (SoftEdge Acquisition System and IonWizard, IonOptix).

### Data analysis

Data analysis methodologies for biomechanical studies are described above. All other quantitative data are presented in tables as mean ± standard deviation (SD), unless otherwise indicated. Differences between two groups were analyzed by Student's *t* test or chi-squared test. For comparisons among more than two groups, ANOVA was performed, followed by *post hoc* Bonferroni correction for multiple comparisons.

### Prior presentations of data

This work was presented orally at the 2006 Scientific Sessions of the American Heart Association (November 2006) and the 2007 Keystone Symposium on Molecular Pathways in Cardiac Development and Disease (January 2007).

## Results

### Tnnt2^+/−^ mice had a mild deficit in transcript and no deficit in protein, with a normal phenotype

**Figure 2:**
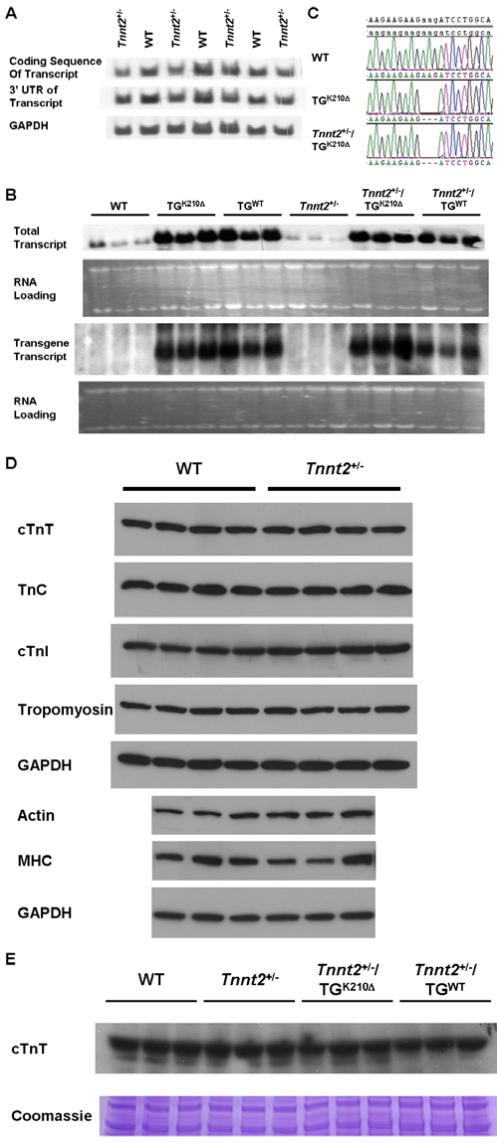
Tnnt2 gene expression in mouse lines. A. Northern blots of total cardiac RNA from wildtype (WT) and *Tnnt2* heterozygous ablated (*Tnnt2* ^+/−^) mice using probes complementary to the coding sequence and the 3′ untranslated region (UTR) of the *Tnnt2* transcript, and the GAPDH transcript as a loading control. A mild deficit in *Tnnt2* transcript, quantified at 18% by densitometry, was apparent in *Tnnt2* ^+/−^ mice. B. Northern blots of total cardiac RNA from mice of the indicated genotypes using a probe complementary to the coding sequence of the *Tnnt2* transcript, comprising both endogenous and transgene transcript (total); and a probe complementary to the human growth hormone 3′ UTR, specific to the transgene transcript. The intensity of 18S and 28S rRNA bands from ethidium bromide stained agarose gels was used to quantify RNA loading. Significant increases in *Tnnt2* transcript were apparent in mice carrying the wildtype (TG^WT^) or the K210Δ *Tnnt2* (TG^K210Δ^) transgene. C. Reverse transcription-PCR and sequencing of cardiac *Tnnt2* mRNA showing deletion of codon AAG encoding lysine at position 210 in hearts from TG^K210Δ^ and *Tnnt2* ^+/−^/TG^K210Δ^, but not wildtype (WT), mice. D. Immunoblots of total protein extracts from the hearts of wildtype (WT) and *Tnnt2* ^+/−^ mice using antibodies specific for cardiac troponin T (cTnT), troponin C (TnC), troponin I (cTnI), tropomyosin, actin, and α myosin heavy chain (MHC). GAPDH loading control immunoblots are shown corresponding to the membranes used for the immunoblots above. Levels of these sarcomeric proteins were unchanged between genotypes. E. An immunoblot of total protein extracts from the hearts of three wildtype, *Tnnt2* ^+/−^, *Tnnt2* ^+/−^/TG^K210Δ^, and *Tnnt2* ^+/−^/TG^WT^ mice was performed using an antibody specific for cardiac troponin T (cTnT). cTnT protein levels were unchanged among all genotypes. Electrophoresis of these protein extracts on a polyacrylamide gel, followed by Coomassie blue staining, was used to correct for the relative quantity of protein loaded for the immunoblot.

Mice lacking one allele of *Tnnt2*, designated *Tnnt2* ^+/−^, were generated by gene targeting (Figure 1). We assessed the effect of loss of one allele on cardiac expression of *Tnnt2*. Northern blots of cardiac RNA from wildtype and *Tnnt2* ^+/−^ mice were hybridized to probes complementary to the coding sequence and the 3′ UTR of the transcript (Figure 2A). Signal intensity showed only a mild decrease in *Tnnt2* transcript, after correction for loading, with both the coding sequence (wildtype, 1.00±0.12; *Tnnt2* ^+/−^, 0.82±0.06; *p* = 0.029; arbitrary units) and the 3′ UTR probe (wildtype, 1.00±0.13; *Tnnt2* ^+/−^, 0.82±0.06; *p* = 0.025). No bands of unexpected size were observed on northern blots in *Tnnt2* ^+/−^ mice, indicating that the mutant allele did not give rise to alternatively spliced transcripts and was in effect a null allele. This deficit in transcript was confirmed by QPCR, which showed that *Tnnt2* ^+/−^ hearts had 0.63±0.01 transcript relative to wildtype (*p*<0.0001). At the protein level, no detectable deficit in cTnT was observed in *Tnnt2* ^+/−^ mice. Immunoblots of cardiac protein showed cTnT levels of 1.00±0.13 in wildtype and 1.01±0.20 in *Tnnt2* ^+/−^ mice (arbitrary units, corrected for total protein loaded, *p* = 0.44) (Figure 2D).

Consistent with the normal quantity of cTnT observed, no phenotype abnormalities were observed in *Tnnt2* ^+/−^ mice at age 8–10 weeks. Echocardiography indicated no differences in left ventricular wall thickness (LVWT), left ventricular end diastolic diameter (LVEDD), and fractional shortening (FS) relative to wildtype mice (Table 1). No histopathological abnormalities, including myofibrillar disarray and fibrosis, were present (data not shown). No arrhythmias were observed on continuous ambulatory ECG recordings. Thus, despite the loss of one allele of *Tnnt2*, *Tnnt2* ^+/−^ mice had only a mild decrease in transcript, no detectable deficit in protein, and a normal cardiac phenotype.

**Table 1:** Cardiac morphology and function of mice assessed by echocardiography at nine weeks age.

	*Tnnt2* ^+/+^ (WT)	*Tnnt2* ^+/−^	TG^K210Δ^	TG^WT^	*Tnnt2* ^+/−^/TG^K210Δ^	*Tnnt2* ^+/−^/TG^WT^
No. of mice	9	13	7	7	6	8
LVWT (mm)	0.76±0.10	0.77±0.14	0.70±0.16	0.74±0.07	0.67±0.17	0.77±0.07
LVEDD (mm)	3.60±0.27 ║	3.62±0.23 ║	3.81±0.26	3.53±0.31 ║	4.21±0.29 *†§#	3.52±0.30 ║
FS (%)	37±7 ║	37±7 ║	29±7 ║#	39±5 ║	18±4 *†‡§#	41±7 ‡║
HR (bpm)	422±41	374±55	390±97	398±47	408±28	397±43

LVWT, left ventricular wall thickness at end diastole; LVEDD, left ventricular end diastolic diameter; FS, fractional shortening; HR, heart rate; bpm, beats per minute; WT, wildtype. Significant differences in means for LVEDD and FS were observed between groups by ANOVA (*p*<0.001). *p*<0.01 by ANOVA and Bonferroni correction versus ^*^WT, ^†^ *Tnnt2* ^+/−^, ^‡^TG^K210Δ^, ^§^TG^WT^, ^║^ *Tnnt2* ^+/−^/ TG^K210Δ^, ^#^ *Tnnt2* ^+/−^/ TG^WT^.

Given the close association of cTnT with other components of the sarcomere, we determined whether loss of one allele of *Tnnt2* was associated with alterations in these other proteins. Relative to wildtype hearts, no changes in levels of troponin C (TnC), troponin I (cTnI), actin, tropomyosin, or β-myosin heavy chain (MHC) were detected in *Tnnt2* ^+/−^ hearts by immunoblot (Figure 2D).

### TG^K210Δ^ mice had mild DCM

TG^K210Δ^ mice were generated carrying a transgene encoding the Lys210 deletion (K210Δ) in *Tnnt2* found in human families with DCM [2]. Similarly, TG^WT^ mice were generated carrying a transgene encoding the wildtype sequence of *Tnnt2*. The TG^K210Δ^ and TG^WT^ lines studied had greater total *Tnnt2* mRNA expression than wildtype mice as assessed by northern blot (Figure 2B). A transgene specific probe showed transgene transcript in only those mice carrying the transgene. Reverse transcription-PCR and sequencing of *Tnnt2* mRNA from TG^K210Δ^ hearts showed a deletion of the codon AAG at nucleotide positions 628–630 (K210Δ) (Figure 2C). In the presence of two similar sequences with unequal abundance, direct sequencing is relatively insensitive to the sequence present at lower frequency. Therefore, our failure to identify wildtype sequence by direct sequence in TG^K210Δ^ hearts suggested that a larger proportion of the transcript was mutant. cTnT protein levels remained unchanged in all transgenic lines. Echocardiography at age 9 weeks demonstrated findings consistent with mild DCM in TG^K210Δ^ compared to TG^WT^ mice (Table 1), with trends towards increased LVEDD (3.81±0.26 vs. 3.53±0.31 mm) and decreased FS (29±7 vs. 39±5%) that did not meet statistical significance. No abnormalities were noted on telemetry ECGs, electrophysiological studies, and histological examination (data not shown).

### Tnnt2^+/−^/TG^K210Δ^ mice had severe DCM

**Figure 3:**
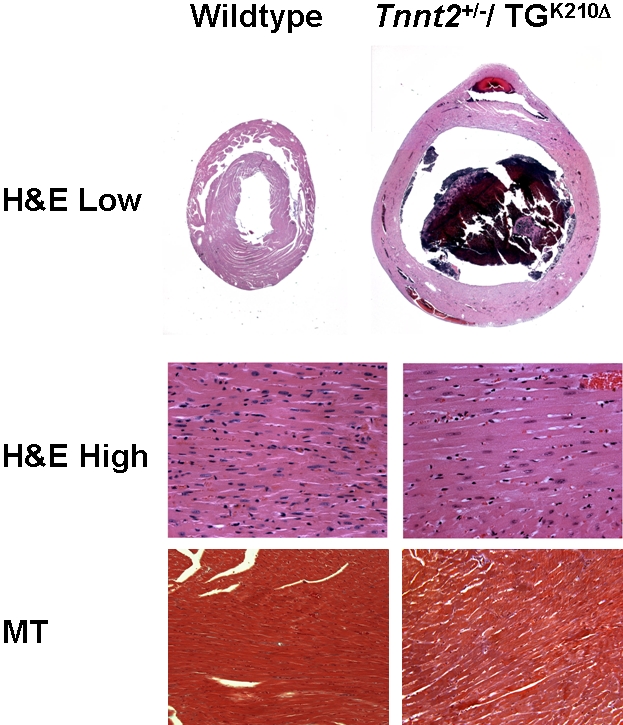
Hematoxylin and eosin staining of hearts from wildtype and *Tnnt2* ^+/^^−^/TG^K210Δ^ mice. Hematoxylin and eosin (H&E) stained tissue is shown at low (2X) and high (10X) magnification, and Masson's trichrome (MT) stained tissue is shown at high (10X) magnification. Massive dilation of the mutant heart was apparent.

**Figure 4:**
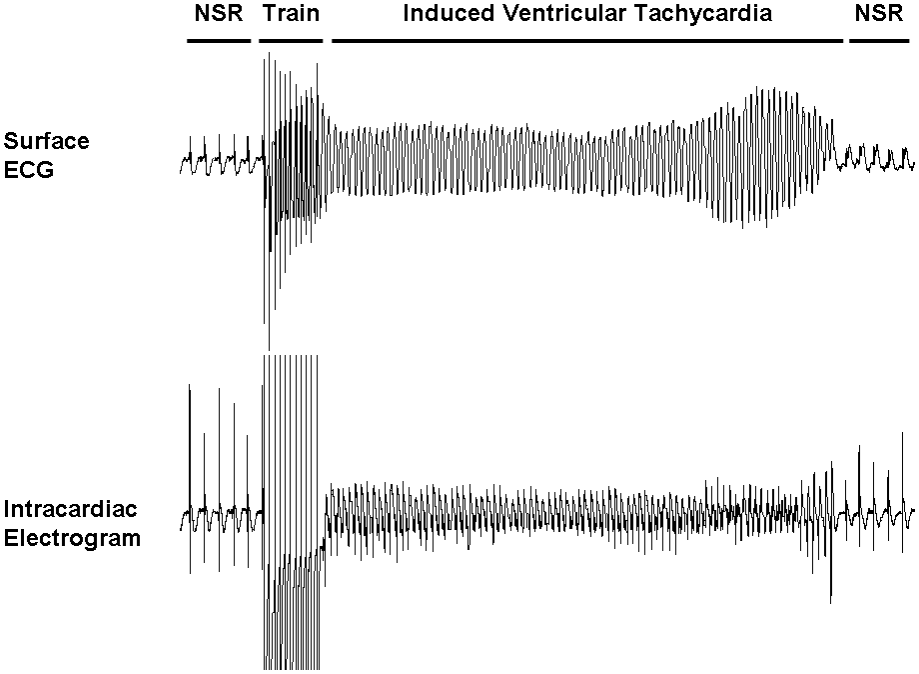
Electrophysiological study in a *Tnnt2* ^+/^^−^/TG^K210Δ^ mouse. A surface electrocardiogram (ECG) and an intracardiac electrogram are shown of a *Tnnt2* ^+/−^/TG^K210Δ^ mouse which had an inducible arrhythmia. Ventricular tachycardia (VT) was inducible by a drive train at 80 ms cycle length, which self-terminated after approximately four seconds. NSR, normal sinus rhythm.

When the mutant and wildtype transgenes were introduced into *Tnnt2* ^+/−^ to generate *Tnnt2* ^+/−^/TG^K210Δ^ and *Tnnt2* ^+/−^/TG^WT^ mice, the *Tnnt2* ^+/−^/TG^K210Δ^ mice recapitulated severe DCM features observed in humans with the K210Δ mutation [2]. As in TG^K210Δ^ mice, reverse transcription-PCR amplification and sequencing of *Tnnt2* mRNA from *Tnnt2* ^+/−^/TG^K210Δ^ hearts showed only mutant K210Δ sequence (Figure 2C), confirming that a larger proportion of the transcript was mutant. Hearts harvested from *Tnnt2* ^+/−^/TG^K210Δ^ mice showed massive dilation (Figure 3). Echocardiography at age 9 weeks showed an increase in LVEDD in *Tnnt2* ^+/−^/TG^K210Δ^ relative to *Tnnt2* ^+/−^/TG^WT^ mice (4.21±0.29 vs. 3.52±0.30 mm, *p*<0.01), and a decrease in FS (18±4 vs. 41±7%, *p*<0.01) (Table 1). Moreover, the phenotype of *Tnnt2* ^+/−^/TG^K210Δ^ mice was more severe than that of TG^K210Δ^ mice with significantly greater impairment in contractility (FS, 18±4 vs. 29±7%, *p*<0.01), along with a trend towards greater left ventricular dilation (LVEDD, 4.21±0.29 vs. 3.81±0.23 mm) that did not meet statistical significance. Electrophysiological studies induced nonsustained ventricular tachycardia at low threshold in two of five *Tnnt2* ^+/−^/TG^K210Δ^ mice as contrasted with only one of 10 wildtype mice (*p* = 0.02) (Figure 4). In addition, *Tnnt2* ^+/−^/TG^K210Δ^ mice relative to wildtype mice had a significantly (*p*<0.05) prolonged QRS duration (24±2 vs. 20±1 ms), HV interval (12±1 vs. 9±1 ms), and QT interval (49±2 vs. 44±2 ms), suggesting conduction delays and repolarization abnormalities.

Molecular changes consistent with heart failure were evident by QPCR at age 50 weeks in *Tnnt2* ^+/−^/TG^K210Δ^ relative to *Tnnt2* ^+/−^/TG^WT^ mice. Atrial natriuretic peptide (ANP), brain natriuretic peptide (BNP), and β-MHC (myosin heavy chain) were all elevated 5.94±1.30, 3.79±1.42, and 9.21±0.68 fold, respectively (*p*<0.01). Increases in collagen I (2.08±0.03 fold, *p*<0.01) and matrix metalloproteinase 2 (MMP2) (1.45±0.06 fold, *p*<0.01) suggested a role for extracellular matrix remodeling in the development of DCM. We observed downregulation of PGC1α 0.13±0.02 (*p*<0.01) and mitochondrial cytochrome B 0.32±0.03 fold (*p*<0.05), suggesting impaired mitochondrial biogenesis.

### The difference in phenotype severity between TG^K210Δ^ and Tnnt2^+/−^/TG^K210Δ^ mice correlated with a difference in the proportion of mutant to endogenous wildtype Tnnt2 gene expression

We performed QPCR to determine whether the differences in the severity of the phenotype in TG^K210Δ^ and *Tnnt2* ^+/−^/TG^K210Δ^ mice may be related to the relative abundance of mutant *Tnnt2* transcript. The relative ratio of mutant to endogenous wildtype transcript was 2.42±0.08 greater in *Tnnt2* ^+/−^/TG^K210Δ^ mice relative to TG^K210Δ^ mice (*n* = 5 each group, *p* = 0.03), suggesting that the presence of a greater proportion of mutant cTnT in *Tnnt2* ^+/−^/TG^K210Δ^ mice leads to a more severe phenotype.

### DCM in Tnnt2^+/−^/TG^K210Δ^ mice is associated with myofiber calcium desensitization

**Figure 5:**
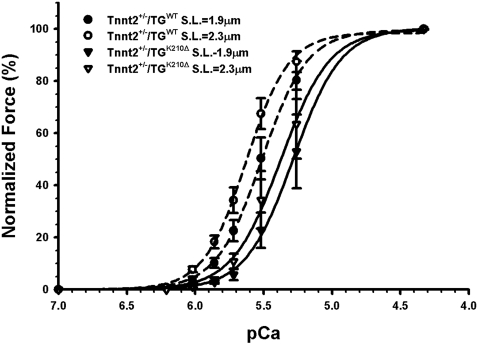
Normalized force-pCa relationships in *Tnnt2* ^+/^^−^/TG^WT^ and *Tnnt2* ^+/^^−^/TG^K210Δ^ skinned papillary muscle fibers. Normalized force (i.e., ratio of force at a given pCa and maximally activated force at pCa = 4.33) developed at a range of Ca^2+^ concentrations was assessed at sarcomere lengths 1.9 µm (*Tnnt2* ^+/−^/TG^WT^ •, *Tnnt2* ^+/−^/TG^K210Δ^ ▪) and 2.3 µm (*Tnnt2* ^+/−^/TG^WT^ ○, *Tnnt2* ^+/−^/TG^K210Δ^ □). There was a rightward shift of the force-pCa curve in *Tnnt2* ^+/−^/TG^K210Δ^ muscle, indicating Ca^2+^ desensitization. Values are mean±SE (*n* = 10 for *Tnnt2* ^+/−^/TG^WT^ and *n* = 7 for *Tnnt2* ^+/−^/TG^K210Δ^).

To determine the cellular basis for reduced cardiac contractility, biomechanical studies were performed on skinned papillary muscle fibers from *Tnnt2* ^+/−^/TG^K210Δ^ and *Tnnt2* ^+/−^/TG^WT^ mice. *Tnnt2* ^+/−^/TG^ K210Δ^ fibers showed Ca^2+^ desensitization relative to *Tnnt2* ^+/−^/TG^WT^ (pCa_50_ = 5.34±0.08 vs. 5.58±0.03 at SL = 1.9 µm, *p*<0.01; 5.46±0.04 vs. 5.71±0.03 at SL = 2.3 µm, *p*<0.01) (Table 2). The length-dependent increase in Ca^2+^ sensitivity (Frank-Starling mechanism commonly seen in cardfoiac muscle) was not different between the two groups of mice (ΔpCa_50_ = 0.13±0.02 for *Tnnt2* ^+/−^/TG^WT^ and ΔpCa_50_ = 0.12±0.05 for *Tnnt2* ^+/−^/TG^K210Δ^, *p* = 0.86) (Figure 5). Moreover, no difference in maximally activated force was detected (Table 2). There was a tendency for the Hill coefficient to increase in the *Tnnt2* ^+/−^/TG^ K210Δ^ fibers; however, this increase was not statistically significant. An increase in Hill coefficient would imply increased cooperativity; however, the biological significance of changes in the Hill coefficient in the skinned fiber model remains poorly understood.

**Table 2:** Characteristics of the force-pCa relationships in skinned papillary muscle fibers.

	pCa_50_	Hill Coefficient	Maximal Force (mN mm^−2^)	pCa_50_	Hill Coefficient	Maximal Force (mN mm^−2^)
Sarcomere Length	1.9 µm	1.9 µm	1.9 µm	2.3 µm	2.3 µm	2.3 µm
*Tnnt2* ^+/−^/TG^WT^ (n = 10)	5.58±0.03	6.54±0.89	45.9±4.6	5.71±0.03	3.65±0.11	62.3±6.5
*Tnnt2* ^+/−^/TG^K210Δ^ (n = 7)	5.34±0.08	9.34±2.47	34.5±5.3	5.46±0.04	6.72±1.09	63.1±10.9
*p*	<0.01	NS	NS	<0.01	NS	NS

pCa_50_, pCa required for generation of 50% of maximal force. *n*, number of skinned fibers studied, taken from a total of 6 hearts in each group. NS, not significant. Data: mean±SE. *p* values correspond to the comparison between *Tnnt2* ^+/−^/TG^WT^ and *Tnnt2* ^+/−^/TG^K210Δ^ fibers at the same sarcomere length.

### The effect of absence of cTnT on embryonic cardiac development

We studied the consequences of complete loss of *Tnnt2* by crossbreeding *Tnnt2* ^+/−^ males and females to generate *Tnnt2* ^−/−^ homozygous null embryos. No newborn *Tnnt2* ^−/−^ pups were observed, suggesting embryonic lethality. Embryos were harvested at 8.5–12.5 days postcoitum (pc). At 11.5–12.5 days pc, 25% of the embryos were dead and in various stages of resorption. All dead embryos which were successfully genotyped were *Tnnt2* ^−/−^. All living and grossly normal embryos were wildtype (*Tnnt2* ^+/+^) or *Tnnt2* ^+/−^. At 9.5 days pc, the genotypes of 76 embryos were 9 *Tnnt2* ^−/−^ (12%), 46 *Tnnt2* ^+/−^ (60%), and 21 *Tnnt2* ^+/+^ (28%), significantly different from the expected Mendelian ratio (*p* = 0.03), with an under-representation of *Tnnt2* ^−/−^ embryos. Thus, some *Tnnt2* ^−/−^ embryos may be lost even earlier than 9.5 days pc.

**Figure 6:**
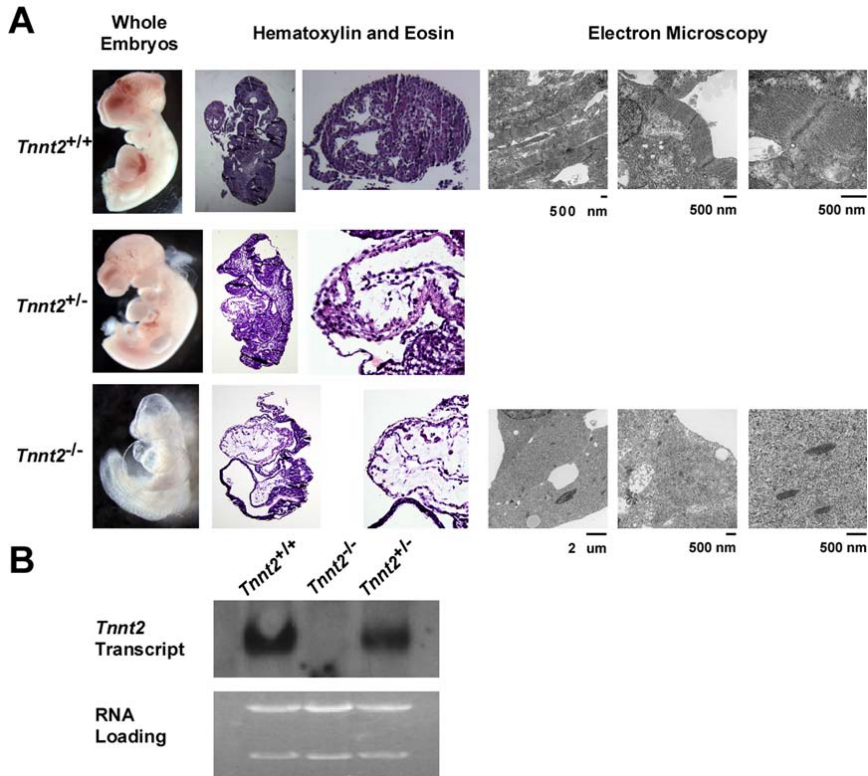
Morphology of *Tnnt2* ^+/+^ (wildtype), *Tnnt2* ^+/^^−^, and *Tnnt2* ^−/−^ embryos. A. Column 1, representative embryos at age 9.5 days postcoitum. Columns 2–3, whole embryos and hearts stained with H&E at low and high power respectively. Columns 4–6, transmission electron microscopy of hearts at increasing magnifications. Whereas *Tnnt2* ^+/−^ embryos appeared normal, *Tnnt2* ^−/−^ embryos showed pericardial effusions on gross inspection, thinning of the myocardium on H&E histology, and loss of organized sarcomeres on electron microscopy. B. A northern blot of total pooled RNA from five embryos of each genotype (*Tnnt2* ^+/+^, *Tnnt2* ^−/−^, and *Tnnt2* ^+/−^) hybridized with a probe complementary to the *Tnnt2* transcript showed absence of transcript in *Tnnt2* ^−/−^ embryos and a 29% deficit of transcript in *Tnnt2* ^+/−^ relative to *Tnnt2* ^+/+^ embryos.

A northern blot on pooled RNA from five embryos of each genotype showed the absence of *Tnnt2* transcript in *Tnnt2* ^−/−^ embryos, and a 29% reduction in *Tnnt2* transcript in *Tnnt2* ^+/−^ embryos relative to *Tnnt2* ^+/+^ embryos (Figure 6B). Whereas *Tnnt2* ^+/+^ and *Tnnt2* ^+/−^ embryos appeared normal, all *Tnnt2* ^−/−^ embryos displayed impaired growth and cardiac abnormalities (*p* = 10^−5^). *Tnnt2* ^−/−^ embryos had appropriately looped hearts. However, the ventricular walls appeared much thinner than those of *Tnnt2* ^+/+^ and *Tnnt2* ^+/−^ embryos and large pericardial effusions suggested heart failure (Figure 6A). On electron microscopy, no organized sarcomeres were visible in *Tnnt2* ^−/−^ hearts. Structures were visible which appeared to be Z bands and possibly disorganized thick filaments. No contractility was observed in any *Tnnt2* ^−/−^ hearts, either spontaneously or on electrical stimulation.

## Discussion

In this study, we ablated the murine *Tnnt2* gene by homologous recombination mediated gene targeting. In the heterozygous state (*Tnnt2* ^+/−^), there was a mild decrease in *Tnnt2* mRNA, with no detectable decrease in total cTnT protein, and a normal cardiac phenotype. Homozygous null mice (*Tnnt2* ^−/−^) were not viable beyond embryonic day 10.5. These embryos demonstrated normal cardiac tube looping, but had thin ventricular walls and apparent heart failure. Electron microscopy of cardiac myocytes showed a lack of organized sarcomeres. When a *Tnnt2* cDNA transgene with a human DCM mutation, Lys210 deletion (K210Δ), was introduced into mice retaining two endogenous alleles of *Tnnt2* (TG^K210Δ^), they exhibited mild DCM, trending towards left ventricular dilation and impaired contractility. When this transgene was introduced into heterozygous null *Tnnt2* ^+/−^ mice lacking one endogenous allele of *Tnnt2* (*Tnnt2* ^+/−^/TG^K210Δ^), they displayed findings typical of severe DCM, including significant left ventricular dilation, poor contractility, conduction delays, repolarization abnormalities, and inducible ventricular tachyarrhythmias. Poor contractility was found to correlate with cardiac Ca^2+^ desensitization in skinned fibers. The difference in severity of DCM in these mice was correlated with the ratio of mutant to wildtype *Tnnt2* transcript.

These results confirm and extend previous data suggesting that mutations in genes encoding sarcomeric proteins do not cause cardiomyopathy by means of haploinsufficiency. Since most HCM and DCM mutations in human families are transmitted in an autosomal dominant pattern, affected individuals carry one normal and one mutant allele. Two mechanisms may lead to cardiomyopathy—inactivation of an allele (a null allele), leading to a reduction in transcript and functional protein (haploinsufficiency); or production of a mutant protein (“poison peptide”) which interferes with normal function (dominant negative) or assumes a new function. In the present study, loss of one allele of *Tnnt2* did not cause HCM or DCM, suggesting that neither cardiomyopathy results from haploinsufficiency. However, severe DCM developed with the addition of a transgene encoding a DCM mutation, suggesting that the mutant transgene encodes a protein with dominant negative activity.

The ablation of one allele of *Tnnt2* in *Tnnt2* ^+/−^ mice led only to a mild 18–37% deficit in *Tnnt2* mRNA, and no detectable decrease in cTnT protein levels. Presumably, the lack of one allele of *Tnnt2* is mitigated by increased wildtype allele transcription or decreased degradation of wildtype transcript; similarly, to compensate for the mild deficit in transcript, increased translation and/or decreased protein degradation must be responsible. Moreover, the levels of other sarcomeric proteins closely associated with cTnT, namely troponin C (TnC), troponin I (cTnI), tropomyosin, actin, and α myosin heavy chain (MHC) were unchanged in *Tnnt2* ^+/−^ mice. In previously reported heterozygous knockout mice lacking single alleles of α**-**tropomyosin and cardiac myosin binding protein C, mRNA levels were approximately half those of wildtype mice, but protein levels remained unchanged [3]–[5]. Thus, stoichiometric ratios of proteins are tightly regulated in the sarcomere.

The complete absence of cTnT is lethal during embryogenesis. This study is the first to detail the effect of ablation of a sarcomeric protein on mammalian embryonic cardiac development. We have shown that cTnT is critical not only to sarcomere function but also sarcomere assembly and structure. Electron microscopy of *Tnnt2* ^−/−^cardiac myocytes demonstrated a complete lack of thin filaments (Figure 6A), although Z bands and thick filaments may possibly be present. Given the lack of organized sarcomeres, it is not surprising that contractile activity was absent in the hearts of *Tnnt2* ^−/−^ embryos. Null mutations in cardiac troponin T in zebrafish [26] and troponin T in the flight muscle of *Drosophila melanogaster* [27] similarly cause a lack of organized sarcomeres. In *Drosophila*, the ablation of myosin heavy chain in the presence of some troponin T mutations restores the normal morphology of the thin filaments and the Z-discs, suggesting that the lack of organized sarcomeres with troponin T mutations alone may be mediated by aberrant actin-myosin interactions.

Whether hemodynamic forces and shear stresses resulting from blood flow are required for normal early cardiac development is controversial. Studies of Na^+^-Ca^2+^ exchanger (*Ncx1*) knock-out embryos, in which some hearts appear not to contract but undergo normal early morphogenesis, suggest that flow is not required. However, a cardiac-specific knock-out [28] and RNA interference [29] of *Ncx1* in neonatal cardiac myocytes demonstrated no effect on contraction. Our studies using cTnT, a direct participant in the contractile apparatus, show that early cardiac tube morphogenesis and looping appear to occur normally, in the absence of contractile activity.

When mutant K210Δ cTnT was introduced by means of a transgene, mice developed DCM. Interestingly, the extent of endogenous *Tnnt2* transcript correlated with the severity of the phenotype. The mutant transgene led to a milder phenotype when both alleles of the endogenous murine *Tnnt2* remained intact, but a more severe phenotype in *Tnnt2* ^+/−^ mice lacking one allele of endogenous *Tnnt2*. We have shown that this phenomenon reflects a dose effect of the ratio of mutant to wildtype transcript, so that greater amounts of mutant transcript, and presumably mutant protein, lead to a more severe phenotype. The mutant cTnT protein likely incorporates into the sarcomere along with a smaller amount of wildtype protein and functions abnormally, leading to DCM. Previous studies of other proteins have similarly found that the severity of cardiomyopathy is related to the ratio of mutant to wildtype protein [30]. Interestingly, a similar knock-in murine model of the *Tnnt2* K210Δ mutation has just been reported by Du and colleagues [31]. Homozygous mutant knock-in mice were found to have a more severe DCM phenotype than heterozygous knock-in mice, suggesting a gene dosage effect. The mutant to wildtype transcript or protein ratio was not measured in the heterozygous mice. It is interesting that not only the homozygous knock-in mice, but even the heterozygous knock-in mice, reported by Du and colleagues had a more severe phenotype than the TG^K210Δ^ described in this report. It is possible that this difference is related to a greater mutant to wildtype cTnT ratio in the heterozygous knock-in mice than the TG^K210Δ^ mice. Alternatively, strain differences may be responsible for the differences in severity of the phenotype.

The phenotype heterogeneity observed in human cardiomyopathy patients with identical mutations may be related to the ratio of mutant to wildtype transcript and protein, which may be a product of modifier genetic, epigenetic, transcriptional and post-transcriptional processes. However, this hypothesis remains to be tested in human subjects.

The precise mechanisms whereby abnormal cTnT protein leads to DCM will require further study. We have demonstrated Ca^2+^ desensitization in *Tnnt2* ^+/−^/TG^K210Δ^ mice, without any changes in maximally activated force or length-dependent activation characteristics (Frank-Starling mechanism). Although normal maximal force generation was not impaired, it is likely that at physiological intracellular Ca^2+^ concentrations the observed Ca^2+^ desensitization leads to decreased force generation *in vivo*. Ca^2+^ desensitization has been observed in permeabilized rabbit cardiac muscle fibers and isolated myocytes into which K210Δ or R141W (another DCM mutation) mutant cTnT was introduced [15]–[18]. The knock-in *Tnnt2* K210Δ mice reported by Du and colleagues were similarly found to have significantly lower Ca^2+^ sensitivity in force generation, which was mitigated by a positive inotropic agent, pimobendan, which directly increases myofilament Ca^2+^ sensitivity [31]. Similar to our findings, these mice retained normal maximal force generation. In mice with DCM mutations in cardiac α-myosin heavy chain (S532P and F764L), we found that contractile function of isolated myocytes was depressed, both mutant myosins exhibited reduced ability to translocate actin but similar force-generating capacities, and actin-activated ATPase activities were reduced [32]. In contrast, some but not all studies have demonstrated an increase in Ca^2+^ sensitivity resulting in increases in force generation, ATPase activity, and hypercontractility in HCM mutations [1], [33]. These findings suggest a paradigm in which DCM mutations lead to Ca^2+^ desensitization and impaired motor function, whereas HCM mutations lead to Ca^2+^ hypersensitization and supernormal motor function.

A potential limitation of this study is that, because the human mutation consists of the deletion of one of four consecutive lysine residues, the mutant and wildtype cTnT proteins could not be differentially quantified in these transgenic mice. Nevertheless, the differences in the relative abundances of mutant and wildtype *Tnnt2* transcripts correlated closely with the severity of the phenotype.

Our data lead us to make the following conclusions. Absence of one *Tnnt2* allele leads to only a mild deficit in transcript and no detectable deficit in protein, and is associated with a normal phenotype. DCM secondary to the *Tnnt2* K210Δ mutation, and likely other sarcomeric protein mutations, results from abnormal function of a mutant protein and is associated with myocyte Ca^2+^ desensitization *in vivo*. The severity of DCM is related to the ratio of mutant to wildtype *Tnnt2* transcript. Haploinsufficiency of *Tnnt2* appears not to be mechanistically related to the development of HCM or DCM. Although cTnT is essential for sarcomere formation and contractility, normal early cardiac morphogenesis and looping of the embryonic heart occurs in the absence of contractile activity.
